# Complications of COVID-19 on the Central Nervous System: Mechanisms and Potential Treatment for Easing Long COVID

**DOI:** 10.14336/AD.2023.0312

**Published:** 2023-10-01

**Authors:** Zhuang-Yao D Wei, Ketty Liang, Ashok K Shetty

**Affiliations:** ^1^Institute for Regenerative Medicine, Department of Cell Biology and Genetics, Texas A&M University Health Science Center School of Medicine, College Station, TX, USA; ^2^Sam Houston State University College of Osteopathic Medicine, Conroe, TX, USA

**Keywords:** Cognitive and mood impairments, Long COVID, Neurological complications, Neuropathological changes, Neuroinflammation, SARS-COV-2, Systemic inflammation

## Abstract

Severe acute respiratory syndrome coronavirus 2 (SARS-CoV-2) invades human cells by binding to the angiotensin-converting-enzyme-2 (ACE-2) using a spike protein and leads to Coronavirus disease-2019 (COVID-19). COVID-19 primarily causes a respiratory infection that can lead to severe systemic inflammation. It is also common for some patients to develop significant neurological and psychiatric symptoms. The spread of SARS-CoV-2 to the CNS likely occurs through several pathways. Once spread in the CNS, many acute symptoms emerge, and such infections could also transpire into severe neurological complications, including encephalitis or ischemic stroke. After recovery from the acute infection, a significant percentage of patients develop "long COVID," a condition in which several symptoms of COVID-19 persist for prolonged periods. This review aims to discuss acute and chronic neurological problems after SARS-CoV-2 infection. The potential mechanisms by which SARS-CoV-2 enters the CNS and causes neuroinflammation, neuropathological changes observed in post-mortem brains of COVID-19 patients, and cognitive and mood problems in COVID-19 survivors are discussed in the initial part. The later part of the review deliberates the causes of long COVID, approaches for noninvasive tracking of neuroinflammation in long COVID patients, and the potential therapeutic strategies that could ease enduring CNS symptoms observed in long COVID.

## 1. Introduction

The world has been inflicted with the Coronavirus Disease-2019 (COVID-19) pandemic since December 2019, which is caused by a novel coronavirus called severe acute respiratory syndrome coronavirus 2 (SARS-CoV-2). COVID-19 was first found in a group of patients suspected of contracting the virus from a wholesale seafood market in Wuhan, China [1]. The first confirmed case of COVID-19 was found on January 29, 2020, in the United States [2]. SARS-CoV-2 was first isolated from three patients at the Wuhan Jinyintan Hospital who presented with severe pneumonia [1]. SARS-CoV-2 is a member of the coronavirus family, typified by enveloped positive-sense single-stranded RNA viruses [3]. Many viruses in the same family can infect the respiratory and enteric systems, liver, or central nervous system (CNS). For example, SARS-CoV-1 can cause similar symptoms as COVID-19 [4]. SARS-CoV-1 caused an epidemic of atypical pneumonia in China in 2002 and spread to other regions in Asia, North America, and Europe [5]. Middle Eastern respiratory syndrome coronavirus (MERS-CoV) is another virus like SARS-CoV-2 that emerged in 2012 [6], and MERS-CoV infection leads to multisystemic symptoms, the most lethal component being pneumonia. MERS also can invade the CNS, like SARS-CoV-1 [7]. Genomic studies of SARS-CoV-1, MERS-CoV, and SARS-CoV-2 showed that SARS-CoV-2 has ~79% genetic similarity with SARS-CoV-1 and ~50% similarity with MERS-CoV [8].

**Figure 1.**
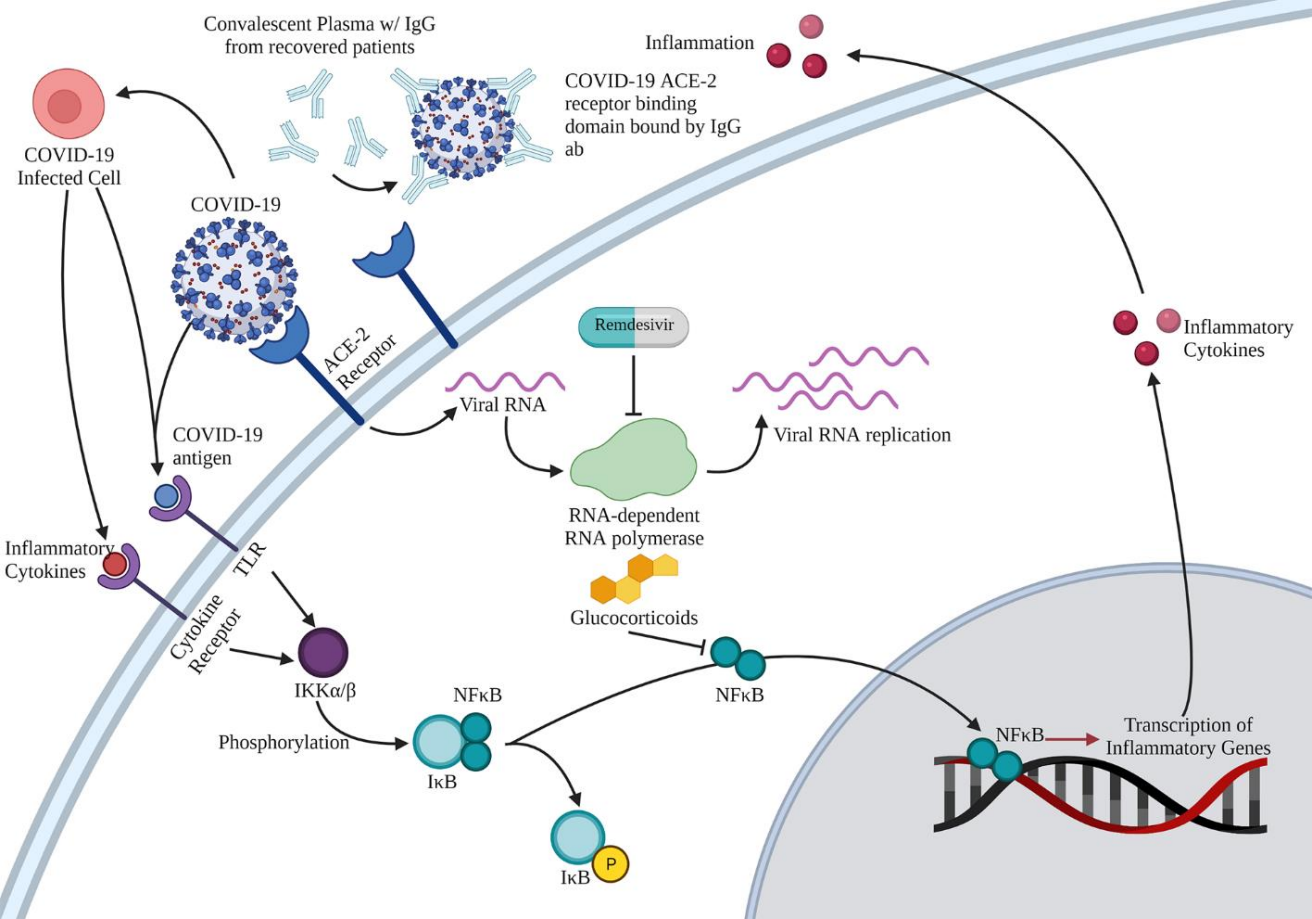
There are two ways that COVID-19 can cause an inflammatory response. COVID-19 antigens can directly bind onto toll-like receptors, or a COVID-19 infected cell can release inflammatory cytokines, which bind to the cytokine receptor on a different cell. These two processes lead to the phosphorylation of NF-kB-IkB, which causes NF-kB to locate into the nucleus and promote the transcription of inflammatory genes. Inflammatory cytokines are then created, which leads to further inflammation of other cells. Convalescent plasma can bind to the COVID-19 ACE-2 receptor binding domain to prevent COVID-19 from entering cells and replicating. If COVID-19 has already entered the cell, the drug remdesivir could be employed to inhibit RNA-dependent RNA polymerase, which can prevent the replication of the virus. Finally, glucocorticoids could be used to block the NF-kB pathway, leading to decreased expression of inflammatory genes.

Patients with COVID-19 most commonly develop fever, cough, and dyspnea, with a possibility of further developing organ damage such as acute respiratory distress syndrome (ARDS), acute kidney injury, cardiac injury, or liver dysfunction [9]. However, some patients who have been diagnosed with COVID-19 have not demonstrated any of these symptoms. Instead, they have initial neurological symptoms such as headache, cerebral hemorrhage, infarction, or other neurological diseases [10]. SARS-CoV-2 seems to infect humans through interactions with angiotensin-converting enzyme-2 (ACE-2). Figure 1 illustrates how SARS-CoV-2 enters cells and subsequent events culminating in an inflammatory response. Areas of high expression of ACE-2 in the brain include nucleus tractus solitarius, nucleus ambiguous, and the area postrema.

SARS-CoV-2 enters cells via binding to ACE-2 with similar efficiency as SARS-CoV-1 [11], with a structural similarity between the respective spike proteins (Fig. 1). The virus is endocytosed into the cell when SARS-CoV-2 binds to the ACE-2 receptor. The RNA viral contents then enter the nucleus for replication and manufacture new viral proteins. These new viral proteins are then matured and released into the surrounding environment, causing inflammation to nearby structures (Fig. 1). The damage caused to cells and organs stems from a T-cell-mediated immune response. Such response involves the entry of SARS-CoV-2 into antigen-presenting cells, such as local macrophages and dendritic cells, through phagocytosis of virus-infected apoptotic cells. The viral antigens are then presented to T-cells in regional lymph nodes, leading to the CD4+ T cell activation of B cells to produce antibodies, and activate CD8+ T cells to kill cells infected by SARS-CoV-2 [12]. The immune response leads to the upregulation of multiple inflammatory and pathogenic cytokines, including interferon-gamma, tumor necrosis factor-alpha (TNF-a), granulocyte-macrophage colony-stimulating factor, and Interleukin-6 (IL-6). These inflammatory mediators are the primary cause of the cytokine storm in COVID-19 patients [13]. Dramatically increased proinflammatory cytokines in different organs lead to organ-specific symptoms. Moreover, the number of regulatory T cells (Tregs) is significantly diminished in COVID-19 patients [14]. Such reduction could weaken the effect of inflammatory inhibition and increase the risk of respiratory failure in COVID-19 patients [14]. Since ACE-2 is also expressed in certain parts of the brain by neurons, neurological symptoms of COVID-19 emerge in a significant percentage of COVID-19 patients.

After recovering from the primary infection and complications, many patients develop lingering neurological or psychiatric effects. This review discusses acute neurological problems associated with SARS-CoV-2 infection and chronic neurological issues after the immune system clears the virus. The review describes how SARS-CoV-2 enters the CNS and causes neuroinflammation, neuropathological changes observed in the post-mortem brains of COVID-19 patients, and cognitive and mood problems in COVID-19 survivors. A significant portion of this review then discusses the incidence, symptoms, and causes of long COVID, similarities between long COVID and chronic fatigue syndrome, means for tracking neuroinflammation in long COVID patients, and potential treatment approaches to combat long COVID symptoms.

## 2. COVID-19 Symptoms

The acute symptoms of COVID-19 are linked to lung infection and/or inflammation. A clinical study of 138 patients with COVID-19 revealed that the majority developed fever, followed by fatigue, dry cough, myalgia, and dyspnea [15]. In the same study, common complications of COVID-19 infection include shock, ARDS, arrhythmias, and acute cardiac injury [15]. Acute venous thromboembolism or pulmonary embolism is another significant complication of a COVID-19 infection that seems to arise even in patients with no predisposing factors for thrombo-embolus formation. Nearly 95% of COVID-19 patients display coagulation disorders typified by increased D-dimer concentration, an extended prothrombin time, and reduced platelet counts [16]. Furthermore, coagulation disorders and thrombosis predict poor outcomes in COVID-19 patients. It has been speculated that endothelial cell activation and dysfunction have a role in causing such clots [16]. Additional neurological symptoms in COVID-19 patients include seizure-like events and new-onset seizures, such as status epilepticus [17-18] and encephalopathy [19-20].

Patients who recover from COVID-19 also have been found to have persistent symptoms after the primary infection [21]. A study following 143 recovered COVID-19 patients revealed that 87.4% reported the persistence of at least one symptom, the most common being fatigue or dyspnea [22]. In addition to the pulmonary and cardiac symptoms, some COVID-19 patients have neurological symptoms. In a survey comprising ~4200 physicians reporting their COVID-19 patients’ neurological symptoms, 95.6% of the physicians recognized at least one neurological symptom in COVID-19 patients [23]. A spectrum of symptoms was observed, the most common being headache, then myalgia, loss of smell and taste, impaired consciousness, psychomotor agitation, lethargy, encephalopathy, cerebrovascular disease, and dizziness. About a third of the surveyed cases reported cerebrospinal fluid (CSF) and electroencephalogram (EEG) abnormalities [23]. The neurological symptoms in COVID-19 patients could result from direct neurological damage induced by SARS-CoV-2, secondary systemic complications, or side effects of drugs employed to treat COVID-19 [24]. Nonetheless, there is a necessity for neurological referral and evaluation for COVID-19 patients [25].

## 3. Mechanisms by which SARS-CoV-2 enters the CNS

**Figure 2.**
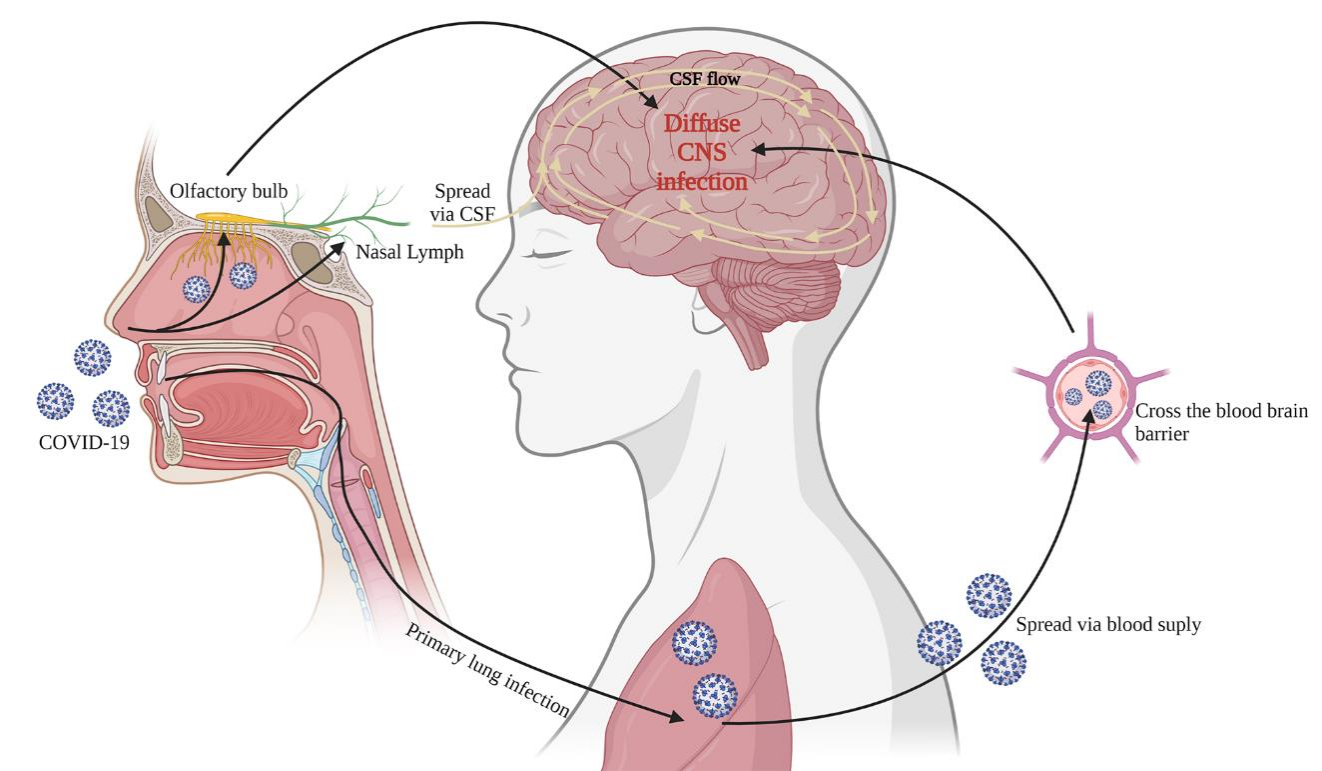
There are multiple ways that COVID-19 can lead to CNS infection. Patients usually get a primary respiratory infection from COVID-19 through inhalation of viral particles or exposure via mucosal surfaces. However, COVID-19 may then spread via the blood supply of the lung and cross the blood-brain barrier, leading to CNS infection. Another way that COVID-19 can spread to the CNS is by crossing the cribriform plate and bind to the ACE-2 receptors expressed by the olfactory bulb. COVID-19 can also enter the brain through the subarachnoid space along the olfactory nerves due to the lymph compartment in the submucosa of olfactory and respiratory epithelia. Once COVID-19 enters the CNS through these proposed mechanisms, CSF flow could play a role in expediting the distribution of viral particles throughout the CNS, leading to a diffuse infection.

There are many hypothesized mechanisms of how SARS-CoV-2 invades the CNS. Figure 2 illustrates the multiple ways by which SARS-CoV-2 likely enters the CNS. Viruses have been demonstrated to enter the CNS using various channels, such as anterograde transport via olfactory nerves into the olfactory bulb and the circulating blood due to blood-brain barrier (BBB) disruption [26-27]. Interestingly, anosmia, the loss of sense of smell, is a common symptom in most COVID-19 patients, supporting the hypothesis that SARS-CoV-2 invades via the olfactory bulb. ACE-2 expression is robust in the olfactory bulb, and SARS-CoV-2 infection has been validated in the human olfactory bulb [28] and in the non-human primate model of COVID-19 [29]. Notably, a recent study using transgenic mice expressing human ACE2 demonstrated that intranasal administration of SARS-CoV 2 resulted in infection of the CNS, which spread mainly from the olfactory bulb [30]. It is important to note that other studies hypothesize that the olfactory route to brain infection is unlikely because the viral entry protein must be expressed abnormally for CNS invasion to occur [31]. Viruses could also enter the brain through the subarachnoid space along olfactory nerves due to the communications between the nasal lymph compartment in the submucosa of olfactory and respiratory epithelia and ethmoidal lymphatics and the brain’s CSF in the subarachnoid space [32-34]. Another way that SARS-CoV-2 could enter the CNS is through the CNS arterial supply and the blood-brain barrier (BBB). Penetration through the BBB is complex due to tight junctions of the endothelial cells, pericytes, and astrocytes. However, BBB dysfunction could occur in systemic inflammatory conditions such as cytokine storms after SARS-CoV-2 infection. COVID-19 may use one or more of these mechanisms, but the ACE-2 receptor is likely essential for transcellular or paracellular migration through the BBB into the CNS [35]. Taken together, ACE2 seems to play a causal role in COVID-19 susceptibility and severity [36]. Another potential avenue of SARS-CoV-2 invasion into the brain is through circumventricular organs. The virus can easily invade the subfornical organ due to a lack of BBB, which can subsequently cause dysfunction of the hypothalamic paraventricular and supraoptic nuclei. The expression of ACE-2 receptors in these nuclei does support this hypothesis [37]. The supraoptic and hypothalamic paraventricular nuclei are principal sources of hormones such as oxytocin and vasopressin and are critical for fluid homeostasis and electrolyte balancing [38]. Disruption and invasion of these nuclei can lead to hydroelectrolytic disorders observed in COVID-19 patients [37].

## 4. Mechanisms underlying CNS Inflammation after SARS-CoV-2 infection

Once SARS-CoV-2 enters the CNS, antigen-presenting cells reporting SARS-CoV-2 antigens trigger a T cell response, leading to a cytokine storm from the subsequent B cell activation and CD8+ T cell activation [12]. The reactionary inflammatory response creates a massive upregulation of proinflammatory cytokines, leading to a hyperactive inflammatory state [39]. Patients with elevated levels of multiple proinflammatory cytokines (IL1B, IL6, IL12, IFNg, IP10, and MCP1) revealed the development of organ systems-related symptoms or multi-organ failure [40]. Elevated proinflammatory cytokines further enhance the density of macrophages, neutrophils, and T cells in the circulation of various organ systems under attack by SARS-CoV-2 [39]. Invasion by immune cells can destroy other cells, destabilizing endothelial cell interactions and damaging vascular barriers [39]. When such immune cell invasion occurs in the CNS, patients are present with various neurological symptoms. Damage to CNS endothelial cells and capillaries leads to a hypercoagulable state in the patients, explaining the 5.7% of COVID-19 patients developing a late cerebrovascular disease, such as an acute ischemic stroke [41]. Hypercoagulability, cytokine storm, increased concentration of antiphospholipid antibodies, and abnormal ferritin levels have been suggested to be underlying causes of ischemic stroke in COVID-19 patients [42]. A study found that COVID-19 patients with elevated levels of D-dimer (a marker for ischemic stroke) and C-reactive protein (CRP) in the blood had a greater propensity for mortality [7]. It was also found that patients with acute SARS-CoV-2 infection and neurological symptoms displayed increased interleukin-12 (IL-12) and interleukin-1b (IL-1b) in the CSF [43]. Recently, it has been hypothesized that SARS-CoV-2 spike protein can act as a pathogen-associated molecular pattern, triggering a significant neuroinflammatory cascade through Toll-like receptor-2 (TLR-2) and TLR4. Such phenomenon is supported by microglia exposed to the S1 subunit of the SARS-CoV-2 spike protein displaying an increased expression of multiple proinflammatory cytokine genes and proteins. Thus, neuroinflammation after SARS-CoV-2 infection can transpire from both the infection/invasion process and the SARS-Co-2 spike protein triggering an inflammatory cascade through TLR signaling [44].

Moreover, in addition to increased production of interleukins, CNS infection by SARS-CoV-2 also stimulates the production of intrathecal antibodies. Since evidence suggests that systemic infection alone is insufficient to elicit the release of intrathecal antibodies, CNS infection in patients could be determined through anti-SARS-CoV-2 antibodies in the CSF [43]. In addition, a monoclonal antibody with specificity for the SARS-CoV-2 spike protein could be isolated from the CSF of a COVID-19 patient but not the peripheral blood. Some patients also demonstrated a high burden of autoreactive antibodies in the CSF, such as antineuronal antibodies. The hippocampus, olfactory bulb, and cerebrovasculature have been shown to contain these autoreactive antibodies. THAP3 and iFT88 are two specific autoantigens identified in the CSF within these regions implicated in anosmia and dystonia [43].

## 5. Neuropathological findings from postmortem brain samples of COVID-19 patients

Postmortem neuropathological studies on COVID-19 patients have reported a variety of findings. Hanley and colleagues found moderate to intense microglial activation in all brain samples. There was also evidence of ischemic changes in the neurons of the cerebral cortex. However, brain tissues did not display signs of necrosis or extensive infiltration of inflammatory cells in the parenchyma or meninges [45]. Roughly 11% of patients examined demonstrated hemorrhagic transformation of cerebral infarction of the middle cerebral artery, likely a complication of SARS-CoV-2 infection [45]. However, there was no evidence of viral genomic RNA in the brain when investigated using qRT-PCR studies [45].

Fabbri and colleagues studied the postmortem brains of 10 COVID-19 patients and found SARS-CoV-2 RNA in the olfactory nerve and brain tissues in 10% of patients, but the olfactory bulb neurons and the tract did not show any histopathological alterations. The COVID-19 patients examined in the study had multiple organ involvement with the quickest time of death, suggesting a hematogenous spread [46]. The macroscopic evaluation showed an edematous surface in all ten brains with widened gyri and meningeal congestion. Histologically, all brains displayed intraparenchymal intravascular microthrombi, perivascular edema, micro-hemorrhages, and hemosiderin-laden macrophages [46]. There was also an indication of a global ischemic injury with eosinophilic neurons in the hippocampal and parahippocampal regions and the cerebellar Purkinje cell layer [46]. Additional studies also reported similar findings, implying that CNS involvement likely worsens the primary respiratory failure [46-47]. The above studies differed in the degree of neuropathological changes, likely due to the small sample size. Therefore, extensive neuropathological studies need to be conducted to understand the degree of CNS involvement after COVID-19 infection and how that can cause long-lasting effects on cognitive and/or mood function or increase the susceptibility to developing neurological and neurodegenerative diseases. Table 1 comprises some of the neuropathological findings found in brain autopsies of COVID-19 patients.

## 6. Cognitive and mood problems in COVID-19 Patients

Some COVID-19 patients with no previous psychiatric history presented delirium and acute psychotic symptoms [48]. In COVID-19 patients with a preexisting psychiatric condition, such as schizophrenia, an acute SARS-CoV-2 infection seemed to precipitate their entry into a psychotic phase [49]. Increasing evidence of psychiatric issues in acute COVID-19 patients underscores the importance of recognizing the multi-systemic ramifications of COVID-19 infection. Patients who recovered from the acute phase of COVID-19 have been found to have lingering cognitive deficits and psychiatric problems. Mcloughlin and associates followed up on 71 COVID-19 patients treated in the hospital using telephone instruments for cognitive status and other delirium assessments. The study demonstrated that hospitalized patients showed a prevalence of delirium with a poor functional outcome that impacted their activities of daily living post-admission [50], implying that delirium could be a significant COVID-19 complication requiring long-term follow-up [50].

**Table 1:** Neuropathological changes in COVID-19.

References	Changes in the Brainstem	Changes in the Midbrain	Changes in the Cerebellum	Changes in the Cerebrum	Other significant findings
Kantonen et al., 2020 [137]	Enlarged perivascular spaces and microhemorrhages.	Alpha synuclein positive Lewy bodies (patient had PD)	Enlarged perivascular spaces and microhemorrhages in white matter.	Enlarged perivascular spaces and microhemorrhages in white matter. Fibrinoid material in cerebral vasculature. Scattered T and B lymphocytes.	---
Jensen et al., 2021 [138]	Brainstem encephalitis of the dorsal medulla.	---	Subacute regional infarct of cerebellar cortex.	Severe multifocal cortical infarction with extensive perivascular calcification and extramedullary megakaryocytes and platelet microthrombi.	---
Bulfamante et al., 2021 [139]	Damaged neurons in medulla with increased number of corpora amylacea in the medulla than in the pons. Marked increase in activated glial elements and GFAP expressing cells. No evidence of hypoxic damage in brainstem.	---	---	---	---
Rhodes et al., 2021 [140]	Acute encephalitis of the medulla.	Midbrain infarcts with significant infiltration of polymorphonuclear neutrophils.	Bilateral cerebellar tonsillar herniations associated with global hypoxic nerve-cell change	Cerebral infarcts with significant infiltration of polymorphonuclear neutrophils.	All cases had acute neutrophilic endotheliitis with variable amount and distribution
Kirschenbaum et al., 2021 [141]	Petechial hemorrhages	Petechial hemorrhages	---	Cerebral petechial hemorrhages	Intracerebral endotheliitis. All cases had evidence of diffuse intravascular thrombosis.
Remmelink et al., 2020 [142]	---	---	---	Cerebral hemorrhage, focal ischemic necrosis and spongiosis	No evidence of viral encephalitis or vasculitis
Fabbri et al., 2021 [46]	Deep seated brainstem infarcts. Microthrombi, ischemic damage, reactive gliosis, microglial activation.	---	Ischemic red neurons in cerebellar Purkinje cells.	Focal microscopic cortical infarcts. Ischemic red neurons in hippocampus and parahippocampal regions.	Intravascular microthrombia and multiple infarcts noted.
Colombo et al., 2021 [143]	Perivascular hemorrhages, gray matter derangement, vacuolization of neuropils. Evidence of astrocytic reaction. Lymphocytic inflammation of vascular walls.	Lymphocytic inflammation of vascular walls.	Loss of neurons in cerebellar Purkinje cell layer. Lymphocytic inflammation of vascular walls.	Destroyed neurons with microglial nodule formation. Microangiopathy characterized by deposits of eosinophilic hyaline material. Lymphocytic inflammation of vascular walls.	---
Younger et al., 2020 [144]	Encephalitis. Localized perivascular and interstitial infiltrates with neuronal cell loss. Axonal degeneration.	---	Encephalitis.	Encephalitis.	---
Normandin et al., 2021 [145]	---	---	Acute hypoxic injury.	Acute hypoxic injury.	Perivascular lymphocytes and focal leptomeningeal inflammation detected.
Ho et al., 2022 [146]					Olfactory axonal damage. Endothelial injury of vasculature.
Martin et al., 2022 [147]				Acute ischemic stroke and intracerebellar hemorrhages	Microhemorrhages/microvascular damage were the most common findings.
Stram et al., 2022 [148]	Brainstem gliosis. Perivascular vacuolization, lymphocytes and microglia. Subtle meningoencephalitis.				Variable white matter gliosis and mineralization.
Ruz-Caracuel et al., 2022 [149]		Neuroinflammation more present in midbrain. Scattered microglial nodules		Small subacute infarcts.	Widespread neuroinflammation and microgliosis with CD68 positive microglia. Global acute hypoxic ischemic changes.

Alemanno and colleagues studied 87 COVID-19 patients after classifying them into different groups based on the type of respiratory assistance they received while admitted to the hospital. These patients underwent neuropsychological evaluation at admission and one month after discharge [51]. Approximately 40% of patients demonstrated mild to moderate depression, and >80% displayed cognitive impairment. However, patients receiving invasive ventilation and sedation (Group 1) had a better cognitive status than the other groups. Interestingly, the patients receiving oxygen therapy through venturi masks (Group 3) showed the most significant difference from Group 1 [51]. Overall, the study implied that patients who received the most invasive respiratory assistance appeared to have a better cognitive function [51]. Since the acute phase of COVID-19 is primarily associated with respiratory complications, more invasive respiratory assistance likely increased the oxygenation and perfusion of the brain, leading to reduced cognitive deficits after recovery.

In another study, 29 patients from 30 to 64 years old who recovered from COVID-19 demonstrated cognitive dysfunction in the sustained attention domain on different neuropsychological tests [52]. The COVID-19 patients were tested with a trail-making test (TMT), sign coding test (SCT), continuous performance test (CPT), and digital span test (DST). No significant differences were seen between the control and the COVID-19 patients in TMT, SCT, or DST. However, in CPT, the correct answers from COVID-19 patients were reduced, which frequently comprised missing numbers and reduced reaction time [52]. Since these studies were done two weeks after the primary infection, additional longitudinal studies need to be done to comprehend long-term cognitive impairments after a COVID-19 infection [52].

## 7. Long COVID or post-COVID-19 syndrome

### 7.1. What is Long COVID?

Long COVID or post-COVID-19 syndrome is a significant post-viral complication in patients who have recovered from an acute COVID-19 infection but still have lingering or continued COVID-19 symptoms beyond the initial period of acute infection and illness [53-54]. Long COVID appears to be a widespread syndrome among COVID-19 patients recovering after hospitalization. The symptoms include anosmia, dysgeusia, breathlessness, chronic cough, extreme fatigue, dyspnea, joint pain, chest pain, palpitations, orthostatic intolerance, memory and attention deficits commonly referred to as “brain fog,” and psychosocial distress typified by anxiety, depression, and insomnia [22, 53, 55-56]. The signs and symptoms are diverse and likely linked to multiple organs and systems. A recent COVID and Cognition (COVCOG) study compared the cognitive function of COVID-19 patients who recovered from infection with individuals who did not contract COVID-19 (N=185/group). The study reported that COVID-19 patients who recovered from infection displayed difficulty concentrating (~78%), brain fog (~69%), and forgetfulness (~68%). A significant percentage of the patients also expressed difficulty returning to work and coping with daily activities. About a third of recovered COVID-19 patients lost their job due to the illness. Interestingly, ~44% of patients had difficulty with medical professionals taking their symptoms seriously [57]. A recent paper characterized six subtypes of long-COVID syndrome due to the multifaceted nature of the syndrome: non-severe COVID-19 multi-organ sequelae, pulmonary fibrosis sequelae, myalgic encephalomyelitis or chronic fatigue syndrome, postural orthostatic tachycardia syndrome, post-intensive care syndrome, and medical or clinical sequela [58]. Long-COVID syndrome has proven to be challenging to study and research because symptoms of the syndrome are also expected from non-infectious causes [59].

### 7.2. Incidence of Long COVID

A study done with 143 patients who recovered from SARS-CoV-2 infection demonstrated that ~87% displayed persistence of at least one symptom, most commonly fatigue [22]. A study in the UK reported that one in five people who tested positive for COVID-19 displayed symptoms lasting 5 weeks or longer, and one in 10 individuals exhibited symptoms lasting 12 weeks or longer [60]. An investigation by Carvalho-Schneider and colleagues showed that, among 150 patients recovering from mostly mild to moderate symptoms of COVID-19 infection, two-thirds displayed at least one symptom persisting at 60 days post-symptom onset, with one-third experiencing the entire spectrum of symptoms as they experienced during the acute episode. The symptoms included anosmia, ageusia, or flu-like symptoms [61]. The symptoms at 30 days post-symptom onset were associated with markers of more severe disease such as hospitalization, oxygen therapy, and dyspnea on presentation in the age group of 40-60 years [61]. The symptoms at 60 days post-symptom onset were linked only for hospitalization in the 40-49 years age group. Xiong and associates reported that ~50% of patients displayed symptoms such as dyspnea and fatigue at ~4 months post-symptom onset [56]. Another study observed 134 COVID-19 patients ~113 days post-discharge from the hospital and found prolonged neurological and psychiatric symptoms such as anxiety, depressed mood, sleep disturbances, and memory impairment [62]. Potential risk factors for developing long-COVID syndrome include female sex, belonging to an ethnic minority, socioeconomic deprivation, smoking, and obesity, emphasizing the complicated nature and pathophysiology of this syndrome [63].

With regards to children, there is not much evidence. However, a study demonstrated that in 129 children diagnosed with COVID-19, 52.7% had at least one symptom 120 days after the diagnosis. Five children developed a severe complication, three had multisystem inflammatory syndrome in children (MIS-C), and two had developed myocarditis [64]. MIS-C is a rare pediatric complication of acute COVID-19 infection, usually presenting 4-6 weeks after the diagnosis. The patient may have high fevers, organ dysfunction, and considerably elevated inflammatory markers. The cause is unknown, but it has many similarities with Kawasaki disease [65]. In adults, MIS-C has been unofficially called Long-COVID [66]. Long-term COVID-19 complications are more common in adults than in children. However, in all cases, long-COVID leads to a decreased quality of life. There are currently over 500 million reported COVID-19 cases, with several North American and European studies estimating an incidence of long COVID of 30 to 90% at 6 months post-diagnosis [54].

### 7.3. Potential causes of varied symptoms in Long COVID

**Figure 3.**
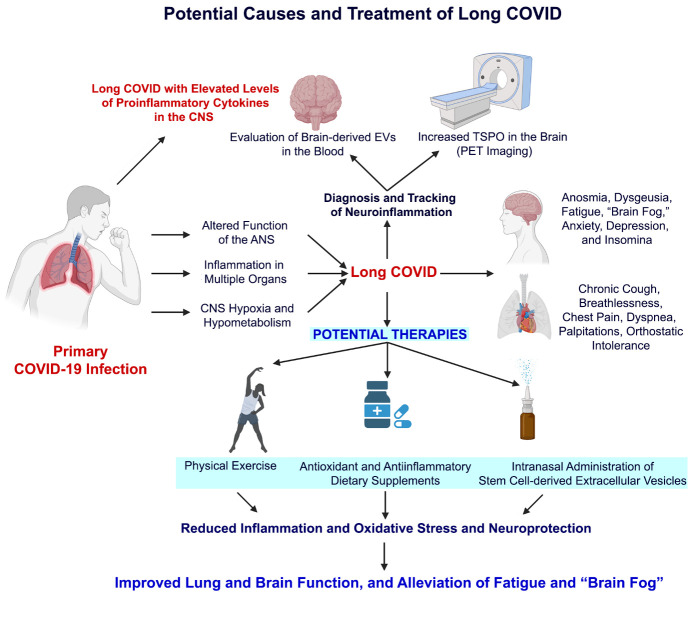
A cartoon depicting potential causes and treatment of Long COVID. Primary COVID-19 infection leads to altered function of the autonomic nervous system (ANS), significant inflammation in multiple organs, and hypoxia and hypometabolism in the central nervous system (CNS). Some of these symptoms continue and evolve into a state of Long COVID with elevated levels of proinflammatory cytokines in the CNS. Significant and persistent neuroinflammation could be diagnosed and tracked by evaluating brain-derived extracellular vesicles (EVs) in the blood and measuring increases in the translocator protein (TSPO) in the brain via PET imaging. The potential therapies for mild to moderate Long COVID could include simple physical exercise and/or antioxidant and antiinflammatory dietary supplements. For severe Long COVID with significant CNS symptoms, intranasal administration of stem cell-derived extracellular vesicles is likely beneficial in improving brain function. The suggested therapies would likely reduce inflammation and oxidative stress, provide neuroprotection, and improve lung and brain function, resulting in alleviating fatigue and “brain fog.”

The precise causes underlying long COVID symptoms are yet to be discerned. Figure 3 depicts the potential causes and symptoms of long COVID. A suggested cause is multiorgan dysfunction due to enduring inflammatory processes in recuperating patients [67]. Indeed, a study has shown that persisting inflammatory response could be seen at 40-60 days post-viral infection in serum samples of COVID-19 survivors [68]. The other potential causes comprise the surfacing of certain conditions post-COVID-19, such as Guillain-Barré syndrome [69], myasthenia gravis [70], and postural orthostatic tachycardia syndrome (POTS) [71]. Furthermore, the long-term psychiatric symptoms after COVID-19 infection may arise due to mechanisms similar to post-traumatic stress disorder [62] and post-stroke depression. As discussed above, patients who received more invasive respiratory assistance appeared to have a better cognitive function [51], demonstrating that CNS hypoxia from COVID-19 likely plays a role in developing lingering cognitive and neurological symptoms. When the brain is hypoxic, many changes occur, such as decreased synaptic signaling, impaired synaptic plasticity, and the release of proinflammatory cytokines [72]. The release of proinflammatory cytokines likely plays a role in the prolonged psychiatric symptoms after COVID-19. Similarly, in post-stroke depression, a link has been observed between increased proinflammatory cytokine levels and a higher prevalence of depression [73]. Furthermore, in mouse model studies, increased levels of IL-1 and TNF-a have been shown to induce depression-like behavior [74].

Prolonged SARS-CoV-2 infection leads to CNS hypoxia due to lung parenchyma invasion and inflammation, which raises the concentration of proinflammatory cytokines in the brain. The longer the infection, the more the brain is exposed to these cytokines. Hence, the patient is more likely to develop long COVID-19 symptoms due to acute neuroinflammation evolving into a state of moderate but chronic neuroinflammation. The occurrence of neuroinflammation and neuronal injury or neurodegeneration observed in the acute phase of COVID-19 suggest that these changes could trigger the development of neurodegenerative diseases such as Alzheimer’s or Parkinson’s diseases in the future [75]. Furthermore, it has been hypothesized that persistent brainstem dysfunction is likely another cause of long-COVID. Such a hypothesis is supported by observations that neural cells in the brainstem express ACE-2 and neuropilin-1, the receptors that facilitate the entry of COVID-19. Studies have also revealed neuropathological changes in the brainstem following COVID-19 infection, as detailed in Table 1. Additionally, long COVID is linked to changes in various brainstem functions, including respiratory, cardiovascular, gastrointestinal, and neurological processes [76]. Another proposed mechanism is hypometabolic dysfunction of various brain regions following acute COVID-19 infection spreading from the nose to the olfactory bulb through the cribriform plate. A study of 35 long-COVID patients with 18F-FDG positron emission tomography (PET) scans revealed hypometabolic activity of the olfactory bulb, limbic/paralimbic regions, brainstem, and cerebellum. On average, these patients had continuous symptoms for three months following their COVID-19 diagnosis [77]. Hypoxia, neuroinflammation, and hypometabolism are closely linked to each other. Indeed, hypoxia and inflammation in the CNS can lead to a hypometabolic state as a defense mechanism [78]. A recent case study of two patients with “brain fog” that had FDG-PET scans done revealed hypometabolic regions in the cingulate cortex. The cingulate cortex is involved in emotions, memory, depression, and decision of action. Interestingly, between these two patients, there was a greater degree of hypometabolism in the patient who experienced more severe COVID-19 [79].

A study reported orthostatic intolerance in COVID-19 survivors in the age range of 26-50 [55], typified by orthostatic hypotension (OH), vasovagal syncope (VVS), and POTS. The authors suggested that the condition is likely due to an abnormal autonomic response to orthostasis (standing up). In a healthy individual, orthostasis increases blood pools in the pelvis and legs and diminishes venous return to the heart. In such a scenario, baroreceptors in the heart and aorta are activated and enhance the sympathetic neural and adrenergic tone via norepinephrine and epinephrine release, leading to tachycardia and vasoconstriction in the splanchnic vascular bed, which increases venous return to the heart. However, in orthostatic intolerance, the release of norepinephrine and epinephrine leads to pronounced tachycardia resulting in palpitations, breathlessness, and chest pain, the common symptoms of long COVID [55]. Also, sustained elevations in catecholamines can induce paradoxical vasodilation, sympathetic activity withdrawal, and vagus nerve stimulation, resulting in hypotension, dizziness, and ultimately syncope or passing out [80]. Thus, orthostatic intolerance in long COVID is likely a result of COVID-19 infection-related altered function of the autonomic nervous system (ANS) [81]. While the precise mechanism by which COVID-19 infection alters the function of ANS is yet to be identified, it is speculated that the generation of autoantibodies against ANS receptors after COVID-19 infection is altering the function of ANS on a long-term basis [55]. Interestingly, a recent study in two patients demonstrated that bilateral anesthetic blockade of the cervical sympathetic chain eased some of their long COVID symptoms. Such observation supports the hypothesis that long COVID is linked to dysautonomia [82].

### 7.4. Similarities between Long COVID and chronic fatigue syndrome

Long COVID also has similarities with chronic fatigue syndrome since both seem to be linked to the altered “glymphatic system” in the brain. As discussed above, a review paper described chronic fatigue syndrome as a possible subtype of the long-COVID syndrome [58]. The glymphatic system is a system of tunnels created by astroglia that remove waste products from the brain and distribute several compounds such as glucose and lipids throughout the brain. The CSF is mainly produced by the choroid plexus [83], which flows through the four ventricles into the subarachnoid space, from where it penetrates the brain parenchyma and exits via the glymphatic system [34, 83]. The glymphatic system facilitates the interchange of CSF and interstitial fluid in the brain parenchyma. Due to pulsatile arteries and CSF pressure gradients, CSF is driven into Virchow-Robin spaces from the subarachnoid space. CSF transport into the brain parenchyma then drives the movement of interstitial fluid towards perivenous spaces, which drains out of the brain into the lymphatic system [83]. This interchange of fluids helps with the excretion of neurotoxins, such as amyloid-beta, as seen in Alzheimer’s disease (AD) [34, 84]. This system also appears to be involved in lipid transport in the brain, sleep modulation, aging, and other neuropathological conditions [83].

As discussed earlier, COVID-19 likely invades and causes inflammation of the cribriform plate and olfactory bulb, potentially blocking the lymphatic and CSF drainage of the brain [85]. The glymphatic system overfills when lymphatic and CSF drainage is backed up, leading to increased intracranial pressure. Studies in mice have shown that ablation of the olfactory sensory neurons has altered CSF drainage through the cribriform plate [86], and other studies have demonstrated increased cranial pressure with an increased block of CSF drainage [85]. The inflammation around the cribriform plate from COVID-19 infection could lead to altered CSF drainage of the brain, which could play a role in Long COVID syndrome. A similar pathology occurs in chronic fatigue syndrome, which is also associated with congestion of the glymphatic system. Chronic fatigue syndrome has very similar symptoms as Long COVID syndrome, including fatigue, pain, sleeping problems, headache, and cognitive impairment [87]. In addition, patients with chronic fatigue syndrome seem to have increased CSF pressure and have had relief of headaches following a lumbar puncture and CSF drainage [88]. Higgins et al. hypothesized that chronic fatigue syndrome might be related to idiopathic intracranial hypertension [88]. Furthermore, a study found that in 229 chronic fatigue syndrome patients, 83% of them had possible signs of intracranial hypertension. Finally, idiopathic intracranial hypertension is also characterized by glymphatic congestion, similar to the proposed mechanism of long COVID discussed above [89].

### 7.5. Feasibility for noninvasive tracking of neuroinflammation in COVID survivors through brain-derived extracellular vesicles in the blood

While it is widely believed that persistent neuroinflammation is one of the major causes of “brain fog” in individuals with long COVID, there is no straightforward diagnostic procedure to evaluate neuroinflammation in COVID-19 survivors. One way to track neuroinflammation noninvasively is through translocator protein (TSPO) PET imaging (Fig. 3). TSPO is in the outer membrane of mitochondria, which is in lower concentration in the brain during healthy states. However, when astrocytes and microglia are activated during neuroinflammatory states, TSPO ligand uptake increases [90]. For example, a study on veterans with Gulf War Illness has assessed neuroinflammation by measuring TSPO through a PET using [^11^C]PBR28, which binds to TSPO [91]. PET imaging of TSPO could also be used to track neuroinflammation noninvasively in long COVID patients to determine the presence or the extent of neuroinflammation. However, such imaging is expensive and likely not available in all medical centers, which is a limitation for discerning the presence and progression of neuroinflammation in millions of COVID survivors presenting one or more persistent symptoms.

Another noninvasive approach to gauge the extent of neuroinflammation is the evaluation of brain-derived extracellular vesicles (EVs) isolated from blood samples (Fig. 3). EVs are membrane-enclosed nanosized vesicles shed by cells carrying a cargo of proteins, miRNAs, and mRNAs [92]. Smaller EVs shed by neurons and glial cells could also be seen in the blood, as they can easily cross the BBB [93]. Because the composition of EVs reflects the physiological or pathological state of cells from which they are shed at the time of release, analysis of EVs derived from specific brain cell types in the blood has been found to be useful for the identification of reliable biomarkers in brain disorders [94-96]. Multiple studies in various neurological and neurodegenerative diseases have revealed that such a liquid biopsy approach helps assess the extent of neuropathology and neuroinflammation in different conditions. The examples include the enrichment of phosphorylated tau (p-tau) at Thr-181 relative to total tau in brain-derived EVs in the blood in the early stages of AD [97-99], elevated levels of cathepsin D, and Lamp-1 proteins in neuron-derived EVs in the blood, ~10 years before the diagnosis of AD [100-101], and increased concentration of alpha-synuclein and neurofilament light chain in patients with Parkinson’s disease (PD) [102-104].

Another study has suggested that periodic characterization of neuron-derived EVs in the plasma of individuals with mild cognitive impairment (MCI) could provide insights on the probability of MCI progressing into AD or dementia [105]. Likewise, the characterization of EVs from dementia patients could help distinguish frontotemporal dementia (FTD) from AD [100]. Furthermore, in a model of Gulf War Illness, analyses of various neuroinflammatory markers in both brain and brain-derived EVs demonstrated that the extent of neuroinflammation could be gleaned from evaluating brain-derived EVs in the blood [106]. Specifically, increased high mobility group box-1 (HMGB1) and proinflammatory cytokine levels in the brain could be seen in neuron-derived EVs in the blood, whereas elevated complement activation-related proteins were apparent in astrocyte-derived cells EVs in the blood [106]. Thus, characterization of disease-related constituents in brain-derived EVs isolated from the blood of COVID-19 survivors could predict the course of neuroinflammation and probability for developing conditions such as dementia or AD. Such characterization of EVs could also help in the application of appropriate treatment for long-COVID.

## 8. Emerging treatment measures for preventing Long COVID

Multiple studies have assessed COVID pathogenesis and identified a few therapeutic approaches for easing long COVID. One pathology is the upregulation of caspases in CD4+ T cells in patients hospitalized with COVID-19 as well as in patients with long COVID. Caspases, involved in the cell death cycle and activation of the inflammatory response, could play a role in the pathophysiology of acute COVID-19 as well as long COVID. An ex vivo study has suggested that emricasan, a pan-caspase inhibitor, could limit the inflammatory response seen in COVID-19. The study has shown that emricasan can bind to the ACE2 receptor and hence can potentially block the entry of the virus into cells [107]. While this finding is interesting, additional in vivo studies are critical to consider emricasan as a potential therapeutic for acute COVID-19 and for preventing long-COVID [107]. Another study suggested that adeno-associated viral (AAV) vectors co-expressing short hairpin RNAs targeted against specific SARS-CoV-2 genes could act as an antiviral agent [108]. In this study, the viral counts in cultured monkey cells and human gut organoids infected with SARS-CoV-2 could be reduced to background levels after administering these virus repressors. Also, in mice infected with SARS-CoV 2, these virus repressors significantly reduced the overall viral load [108]. Another recent study targeted acute COVID-19-induced pulmonary fibrosis, a condition considered a strong predictor for developing long COVID. Acute COVID-19 infection leads to an accumulation of inflammatory myeloid cells, neutrophils, and macrophages, which promotes pulmonary fibrosis by activating the transcription factor JUN and enhancing the activity of CD47 and IL-6 [109]. The study showed that, in a mouse model of COVID-19-induced lung fibrosis, blockade of CD-47/IL-6 can reduce fibrosis, emphasizing CD47 and Il-6 as potential therapeutic targets [109].

## 9. Potential treatment measures for Long COVID patients

A guideline has been developed by the Scottish Intercollegiate Guidelines Network and the Royal College of General Practitioners to help identify and make recommendations for the treatment of Long COVID. The guidelines recommend offering a complete blood count, kidney function test, liver function test, CRP, ferritin, BNP, and thyroid function tests. Additional recommendations include orthostatic blood pressure measurements in patients who display palpitations or dizziness and chest X-ray in patients exhibiting continuing respiratory symptoms for 12 weeks. Furthermore, a psychiatric referral is suggested if there are risks of suicide or self-harm. Overall, a multidisciplinary approach is recommended for the best degree of care for long COVID patients (www.nice.org.uk/guidance/ng188) [60].

“Brain fog” is one of the significant features of long COVID, but its precise cause is unknown. Recently, 150 patients who recovered from COVID-19 underwent comprehensive neuropsychological assessments. Several treatment approaches can potentially reduce mild to moderate neuroinflammation typically associated with “brain fog.” Such moderate neuroinflammation commonly observed during aging is modifiable through lifestyle changes or dietary supplements [110]. For example, simple physical exercise can decrease oxidative stress and positively modulate the balances between pro-and antiinflammatory cytokines in the brain by attenuating TNF-a and IL-1b and upregulating IL-10. Exercise also appears to suppress apoptotic neuronal cell death [111-113]. A recent study in a model of Gulf War Illness has shown that even moderate intermittent physical exercise can reduce neuroinflammation and improve brain function by modulating the proinflammatory microglia into anti-inflammatory phenotypes [114]. Vaso-protective components of the renin-angiotensin system in the periventricular nucleus and rostral ventrolateral medulla were also impacted in regular exercise [111]. Thus, lifestyle changes such as moderate but regular exercise could ease the persistent neuroinflammation, brain fog, chronic fatigue, and orthostatic intolerance observed in survivors of COVID-19 infection (Fig. 3).

Furthermore, over-the-counter supplements could reduce neuroinflammation in COVID-19 survivors (Fig. 3). Indeed, studies have shown that resveratrol can prevent age-related cognitive dysfunction through modulating moderate chronic neuroinflammation [110] and provide neuroprotection after status epilepticus through suppression of oxidative stress acute neuroinflammation [115]. Similarly, curcumin has been shown to improve brain function in a model of Gulf War Illness through reduced neuroinflammation and improved mitochondrial function [114-117]. There have been many studies on the neuroprotective effects of these compounds in models of traumatic brain injury, stroke, PD, and AD [118-119]. The potential mechanisms by which resveratrol suppresses neuroinflammation include inhibition of reactive oxygen species production, suppression of mitogen-activated protein kinase (MAPK) signaling pathway, and suppression of the NF-kB pathway [119]. Glycyrrhizin induces antiinflammatory effects by inhibiting the HMGB1-mediated neuroinflammatory response [120]. Omega-3 fatty acids, also known as n-3 polyunsaturated fatty acids, and their metabolites have been shown to modulate microglia into anti-inflammatory phenotypes [121]. They can also regulate microglia through modulation of signaling pathways or control of gene expression through surface or intracellular receptors. Studies have shown that these metabolites can impair the membrane location of the CD-14 receptor and TLR 4, leading to a decrease in microglia-mediated inflammatory response [121]. Ellagic acid found in red raspberries, strawberries, pomegranates, and walnuts, is another natural antioxidant and antiinflammatory compound. Studies have shown that ellagic acid supplementation can prevent D-galactose-induced cognitive dysfunction in rats through downregulation of Bcl-2 and Bax expression and up-regulation of caspase-3 [122]. Thus, besides physical exercise, over-the-counter dietary supplements with significant antioxidant, antiinflammatory, and neuroprotective properties are likely beneficial to combat the persistent mild to moderate neuroinflammation associated with long COVID.

Combating severe neuroinflammation in patients with long COVID might need more advanced antiinflammatory therapies. One of the emerging approaches is the intranasal administration of EVs generated from mesenchymal stem cells and neural stem cells [123-125]. Studies have shown that intravenous or intranasal administration of stem cell-derived EVs in models of acute TBI and status epilepticus exhibiting severe neuroinflammation resulted in improved brain function with neuroprotection and substantial reductions in neuroinflammation [124-126]. EVs from stem cells exert anti-inflammatory and neuroprotective effects through therapeutic nucleic acids and proteins they carry from parental cells. Since intranasal administration of EVs results in their quick delivery into neurons and glia in virtually all brain regions [123, 125], such a non-invasive approach seems appropriate for treating long COVID associated with significant neuroinflammation. It is also noteworthy that intravenous administration of mesenchymal stem cells or EVs generated from mesenchymal stem cells in acute COVID-19 patients have been found to be safe and helpful in downregulating cytokine storm [127-133]. However, to translate EV therapies for long COVID, a large amount of good manufacturing practice (GMP) grade (clinical grade) EVs with potent antiinflammatory effects need to be generated from stem cells [134].

## 10. Conclusions

COVID-19 primarily is a respiratory infection involving the binding of the SARS-CoV-2 to ACE-2 expressed on cells of the lungs and the respiratory system. Various mechanisms have been proposed on how COVID-19 viral particles from the respiratory system enter the CNS. The potential routes include transport via olfactory nerves passing through the cribriform plate into the olfactory bulb, lymphatics between the nasal mucosa and the subarachnoid space into the CSF, and direct penetration through the BBB into the CNS parenchyma. Once in the CNS, viral particles can bind to ACE-2 receptors with the spike protein to get endocytosed into neural cells, where they can replicate and cause a significant inflammatory response. The inflammatory response can generate a cytokine storm, leading to acute neuroinflammation and subsequent neurological symptoms and complications.

The inflammatory response in the brain causes many neurological symptoms such as headache, dizziness, anosmia, and ageusia [135]. Serious complications have also arisen from CNS inflammation, including stroke, hemorrhage, venous thrombosis, encephalitis, meningitis, and loss of sensation [135]. Neuropathological studies on post-mortem brain tissues from COVID-19 patients correlated many complications with ischemic changes and edema. Such studies varied regarding the extent of neuropathological changes, likely due to smaller sample sizes.

Moreover, a significant percentage of patients recovering from an acute COVID-19 infection have been found to have lingering cognitive problems, decreased ability for concentration, increased anxiety, and a higher propensity for developing depression, among other symptoms related to the respiratory and cardiovascular systems. Individuals exhibiting such lingering symptoms are now recognized as having "Long COVID" or "post-COVID syndrome." The CNS-related symptoms in Long COVID appear to result from persistent mild to moderate neuroinflammation, likely involving the activation of microglia and astrocytes. Non-invasive diagnostic and prognostic approaches need to be developed to assess the occurrence and progression of neuroinflammation in Long COVID cases. Evaluation of brain-derived EVs in the circulating blood could be one of the strategies to quickly assess the extent of neuroinflammation in patients displaying Long COVID. Also, lifestyle changes such as physical exercise and antioxidant/nutritional supplements could improve brain function in Long COVID conditions associated with mild to moderate neuroinflammation. However, Long COVID cases exhibiting severe neuroinflammation might require advanced anti-inflammatory therapies. Intranasal administration of mesenchymal stem cell-derived EVs could be one of the therapies to consider in Long COVID patients with severe neuroinflammation. The ability of EVs to exert potent antiinflammatory and neuroprotective effects in models of severe neuroinflammation supports such a suggestion. COVID-19 is still infecting many unvaccinated people daily and would likely induce long-term neurological complications, possibly in millions of survivors. Therefore, strategies to combat Long COVID are urgently needed to prevent a massive increase in dementia or AD cases from occurring in the coming decade. A recent study has demonstrated that a variant of the oligoadenylate synthetase 1 (OAS1) gene expressed in microglia is involved in the higher risk for AD and severe neurological complications of COVID-19 [136]. Therefore, patients experiencing severe neurological complications of COVID-19 or Long COVID with predominant CNS symptoms need to be tracked and treated appropriately to thwart the progression of symptoms into AD or dementia.
